# Necrological

**Published:** 1880-05

**Authors:** 


### NECROLOGICAL.

> "Death is the crown of life:
> Were death denied, poor man would live in vain."

We regret to learn of the death of Professor Samuel Choppin, M. D., of New Orleans, just as this issue is going to press. Dr. Choppin was a gentleman of noble and generous impulses, and unquestioned gallantry and bravery. He had attained to eminence in his profession, particularly in surgery, to which he devoted much of his attention.

Professor Sayre of New York has been sadly afflicted in the early death of his talented and estimable son. Young Dr. Sayre bade fair to equal his distinguished father as a surgeon and physician. We extend our heartfelt sympathy to the afflicted father and family in their great bereavement.

